# Erectile Dysfunction after Kidney Transplantation

**DOI:** 10.3390/jcm9061991

**Published:** 2020-06-25

**Authors:** Anna Perri, Giulia Izzo, Danilo Lofaro, Sandro La Vignera, Antonio Brunetti, Aldo Eugenio Calogero, Antonio Aversa

**Affiliations:** 1 Kidney and Transplantation Research Center, Department of Nephrology, Dialysis and Transplantation, Annunziata Hospital, 87100 Cosenza, Italy; anna.perri@unical.it (A.P.); danilo.lofaro@unical.it (D.L.); 2 Department of Experimental and Clinical Medicine, Magna Graecia University Catanzaro, 88100 Catanzaro, Italy; giulia_izzo@virgilio.it; 3 Department of Clinical and Experimental Medicine, University of Catania, 95123 Catania, Italy; sandrolavignera@unict.it (S.L.V.); acaloger@unict.it (A.E.C.); 4 Department of Health Sciences, University “Magna Græcia” of Catanzaro, 88100 Catanzaro, Italy; brunetti@unicz.it

**Keywords:** end-stage renal disease, hypogonadism, prolactin, intracavernous injection, phosphodiesterase type-5 inhibitors

## Abstract

Patients with kidney transplantation often have a worse quality of life than the general population. One of the reasons for this, in male patients, is the high prevalence of erectile dysfunction. This is mainly due to the presence of comorbidities, surgery for kidney transplantation, adverse drug effects, psychological changes related to chronic disease, as well as hyperprolactinemia and hypogonadism. Whenever these endocrine dysfunctions occur after kidney transplantation, they must be corrected with appropriate treatment, i.e., testosterone replacement therapy. Administration of the phosphodiesterase-5 inhibitor (PDE5i) sildenafil at the recommended posology does not significantly alter the pharmacokinetics of the calcineurin inhibitors cyclosporin A or tacrolimus and does not impair kidney allograft function. Tacrolimus increases the peak concentration and prolongs the half-life of PDE5i in kidney transplant patients and, therefore, daily administration cannot be recommended due to the significant drop in blood pressure. Intracavernous injection or topical application of alprostadil can be a second-line option for the treatment of erectile dysfunction after kidney transplantation, which does not alter cyclosporine concentrations and does not deteriorate kidney function. Finally, penile prostheses can be successfully implanted following pelvic organ transplantation after eliminating the risk of infection associated with surgery.

## 1. Introduction

Impaired sexual function is very common in end-stage renal disease (ESRD) patients, with a prevalence of 60–90% in both genders [1,2,3]. Although improvement of sexual dysfunction has been reported after kidney transplantation [4,5,6], some studies have shown that this condition can persist even after successful transplantation [7,8].

In a systematic review and meta-analysis of 50 studies, the rate of erectile dysfunction (ED) in patients with chronic kidney disease (CKD) was shown to be 75%, whereas it decreased to 59% in kidney transplantation recipients (KTRs). This suggests that restoration of the glomerular filtration rate (GFR) after transplantation may improve erectile function, although the reduction in ED severity and prevalence might depend on the predominant etiological mechanism [2]. The high rate of ED in KTR patients may be ascribed to various factors, such as long-term uremia, persistent sex hormone abnormalities (decreased testosterone and increased LH, FSH, and prolactin levels), and vascular modifications of penis arteries caused by iliorenal artery anastomosis, the introduction of immunosuppressive drugs, and the high rate of anxiety and depression [9,10]. Furthermore, many KTRs have multiple risk factors for ED, including diabetes and hypertension, while transplantation does not have any effect on these underlying risk factors [11].

The availability of published data that can be used to compare pre- and post-transplantation erectile function, as well as to evaluate the response to treatments, is very limited. Moreover, the main limitations of these studies are their reliance on a small number of patients investigated and the suboptimal or lack of uniform assessment of the outcome measures. In the present article, we perform a Medline search in the attempt to review the most important clinical and experimental lines of evidence on the main factors influencing the onset of ED after renal transplantation or their persistence/improvement after dialysis interruption. Furthermore, because of the paucity of the studies, we aim to highlight the best practice in the treatment of ED in KTRs, underlying that sexual dysfunction is an underestimated topic by nephrologists in both men and women with CKD, also after kidney transplantation.

## 2. Factors Influencing ED after Kidney Transplantation

In KTRs, ED has a multifactorial etiology since either organic, psychological, or mixed abnormalities are involved [12]. Predisposing factors usually include conditions/comorbidities related to CKD [12], which may not always be corrected by transplantation [13,14]. The beneficial effect of kidney transplantation is still controversial [13,14]. While some authors have reported an improvement of ED in KTRs [12,15], others have pointed out a persistent or de-novo onset of ED [4,12,16]. Indeed, the systemic organic damage in patients with CKD, comorbidities, and hemodialysis shows a progressive and irreversible course, which is frequently unresponsive to transplantation and may negatively impact KTRs’ sexual function [16,17].

Dysfunctions of the hypothalamic–pituitary–gonadal (HPG) axis play a crucial role in the pathogenesis of ED (Table 1). In particular, testosterone deficiency may negatively impact on penile structure, function, and hemodynamics, leading to ED [12,17]. Moreover, hormonal abnormalities (Table 1) such as hypogonadism (primary or secondary), hyperprolactinemia, and secondary hyperparathyroidism may improve or persist after transplantation [12]. Besides hormonal dysfunctions, many other preexisting conditions (Table 1) such as diabetes mellitus and metabolic risk factors, autonomic nervous system disturbances, peripheral neuropathy, cardiovascular, and endothelial dysfunctions, or a combination of these disorders, in addition to anemia, secondary hyperparathyroidism, drugs, psychological factors, age, voluptuous habits, and education, may differently impact ED in KTRs [12,18]. In particular, metabolic syndrome and diabetes mellitus are often associated with lower testosterone levels, and may also cause ED through endothelial damage and a multifactorial prothrombotic state, leading to arteriopathy and neuropathy [12,19]. In patients with CKD, several molecular and ultrastructural abnormalities may lead to damage of cavernous tissue and distal penile arteries [16]. Indeed, persistent penile anatomical abnormalities, usually occurring in uremic patients, include changes in smooth muscle cells, which are characterized by the reduction of dense bodies in the cytoplasm, thickening of the basement membrane, interstitial fibrosis (with a reduction in cell-to-cell contacts), thickening and lamination of basement membranes of endothelial cells, and increased accumulation of collagen between nerve fibers [20]. Besides the metabolic disturbances, anemia (Table 1) is also involved in penile morphological and functional damage [20].

Moreover, the duration of hemodialysis before transplantation, a lower diastolic blood pressure, and peripheral arteriopathy can also influence ED in KTRs [4]. After transplantation, age, comorbidities, drugs, vascular conditions, transplant surgical procedures, iterative transplantation, voluptuous habits, interpersonal problems, altered body image perception, and psychological factors (e.g., self-esteem, stress, and depression) may impact on ED (Table 1) [12,16,17]. In particular, age is an important risk factor for both pre- and post-transplantation ED [12], and it is directly correlated with the severity of ED [13]. Tian and colleagues reported a prevalence of ED of 60% in patients <30 years old, 66% in those of 31–40 years; 75% in those of 41–50 years; 87% in those of 51–60 years, and 92% in >60 years [20]. In contrast, a prospective, interventional, nonrandomized study showed that erectile function worsened in KTRs <45 years, whereas it did not change in KTRs >45 years old [16].

Education displays an inverse correlation with ED, which may be explained by less attention to healthcare, quality of life, and sexuality in people with low education [20]. The role of voluptuous habits, such as smoking/drinking, is still unclear. Smoking notoriously shows detrimental effects on erectile function [16]; however, some studies reported no association with ED in KTRs [4,16].

The duration of the hemodialytic treatment, which patients have undergone before kidney transplantation, also affects erectile function in KTRs [14,16,20]. Improvement of ED has been reported in KTRs who underwent dialysis for 6 months, whereas those who were dialyzed for 6–24 months showed no ED improvement. This may probably be due to the effect of early transplantation in preventing/reversing penile morphological/functional and hemodynamic abnormalities, as well as hormonal dysfunctions [14]. Data on the association between ED and professional factors or graft durations are unclear, though some authors have reported no association with erectile function [20]. Although comorbidities such as diabetes mellitus and/or peripheral neuropathy, dyslipidemia, and hypertension are independent risk factors, KTRs sometimes pay particular attention to control these pathologies to delay chronic allograft nephropathy. Therefore, the impact of these factors on ED may be variable [20].

Drugs such as immunosuppressant and antihypertensive agents are involved in the onset of ED in KTRs. In particular, calcineurin inhibitors (CNIs), i.e., cyclosporine, tacrolimus, mTOR inhibitors, as well as corticosteroids, may impact on the endothelial function [13,14,16,17] and/or the testicular function/structure [13].

Surgical procedures during transplantation, such as the interruption of iliac artery distal ends, may sometimes cause insufficiency of penile blood flow, leading to ED [17,20]. The type of arterial anastomosis differently influences the prevalence of ED in KTRs because of the impact on cavernosal artery hemodynamics [21]. Evidence reports an increased ED in KTRs revascularized through end-to-end anastomosis to the internal iliac artery compared to end-to-side anastomosis to the external iliac artery, suggesting a negative role of the unilateral ligation of the internal iliac artery in erectile function following kidney transplantation [21]. Indeed, when the kidney graft is anastomosed end-to-end to the internal iliac artery, the blood inflow from the internal pudendal artery is diverted [21]. However, the prevalence of ED is still high even in KTRs revascularized through anastomosis at the external iliac artery [21], which shows a slight reduction in penile arterial inflow [21]. Moreover, penile hypoafflux and ED may also be worsened or caused by iterative transplantation when the internal iliac artery is more likely used for end-to-side anastomosis [20].

Finally, psychological factors such as self-esteem, altered body image perception, worrying, anxiety, and depression (which are related to kidney disease and transplantation) usually affect erectile functioning [20,22]. Indeed, many symptoms in KTRs may derive from somatic and psychosocial comorbidities persisting after the transplantation procedure [13].

## 3. Impact of Immunosuppressant Drugs on ED

The goals of immunosuppressive therapy in KTRs are to prevent graft rejection and complications and to reduce morbidity. KTRs are maintained on an immunosuppression regimen that includes one to three drugs. The major immunosuppressive agents used in these patients are CNIs, cyclosporin A (CsA) and tacrolimus, mammalian target of rapamycin—mTOR—inhibitors (sirolimus and everolimus), antimetabolites (azathioprine, mycophenolate-mofetil), and glucocorticoids (prednisone) [23].

Nowadays, CNIs constitute the cornerstone of the immunosuppression regimen following kidney transplantation [24]. However, these drugs are known to be nephrotoxic and can cause acute and/or chronic toxicity on the graft [25,26], concomitantly contributing to the new onset of diabetes [27] and hypertension [28]. Growing literature has shown that CNI minimization or conversion to alternative non-nephrotoxic maintenance immunosuppressants, using mTOR inhibitors or the costimulation blocker belatacept, may improve long-term allograft function and survival, reducing the correlated complications [29,30]. The evidence published on the correlation between CNI-based immunosuppressive regimens and ED in KTRs is controversial [4,31,32,33,34]. The study of El-Assmy and colleagues showed that ED was more prevalent among patients receiving CsA, highlighting that CsA may negatively impact penile hemodynamics through different mechanisms, leading to ED [35]. Data on this topic reported that treatment with CsA increases median blood pressure, concomitantly suppressing nitric oxide (NO)-mediated smooth muscle relaxation, essential for erection [36]. Moreover, in endothelial cells, therapeutic doses of CsA, by reducing caveolae cholesterol content, promoted the translocation of eNOS from caveolae, leading to a decreased generation of NO [37,38]. Interestingly, CsA-induced NO modulation, by upregulating TGF-β1 expression, increases matrix deposition and decreases matrix degradation [39]. This effect, concomitantly with a reduction of collagen degradation promoted by CsA exposure in both epithelial and fibroblast cells [40], may induce fibrosis of the corpora cavernosa.

Although vascular pathology represents the key mechanism underlying ED, it has been reported that testosterone deficiency may affect erectile function [41]. Several experimental models have investigated the effects of CsA on spermatogenesis and endocrine function, demonstrating that CsA inhibits the growth and the steroidogenic capacity of rat Leydig cells [42,43], and this effect occurs in a dose-dependent manner [44]. This suggests that CsA-induced hypogonadism may represent a mechanism involved in ED.

Recent advances have elucidated the crucial role of mTOR in the male reproductive potential [45]. However, several studies have shown that mTOR-inhibitors, mainly sirolimus, negatively impact male gonads of KTRs after a few years of treatment. Nevertheless, the underlying molecular mechanisms remain not completely understood [45]. Certainly, male infertility represents the most striking side effect, and sirolimus causes a decrease in sperm count, motility, and viability [46,47,48]. Furthermore, in-vivo studies have revealed that doses of rapamycin equivalent to therapeutic levels affect testicular development and HPG axis function and induce marked histological changes of testicular structures. The last ones include dystrophy of seminiferous tubules, leading to the impairment of spermatogenesis [49,50,51]. Some studies have reported that sirolimus is associated with a decrease in serum total testosterone levels, concomitantly with a rise of LH and FSH, although it is unclear whether sirolimus-related hypogonadism impairs the erectile function [13,52,53,54,55].

No data are available to assess whether everolimus is more or less gonadotoxic than sirolimus. Interestingly, it has been reported that the side effects on spermatogenesis, as well as on sex hormones, may be reversible by switching from the administration of mTOR-inhibitors to other immunosuppressants [49]. Immunosuppressive therapeutic strategy in KTRs frequently includes corticosteroids that significantly reduce acute rejection, but cause several long-term adverse effects, even at low doses. Therefore, some studies have suggested quitting glucocorticoids after kidney transplantation, even if the optimal time point has not yet been established [56]. Glucocorticoids decrease testosterone synthesis directly via gonadal steroid receptors and centrally by acting at the hypothalamic–pituitary level. In-vitro and in-vivo studies have shown that glucocorticoids decrease hypothalamic GnRH release and basal- or GnRH-stimulated release of LH, and reduce testis responsiveness to LH and concentrations of LH receptors [57]. Excessive glucocorticoid exposure suppresses androgen synthesis and promotes Leydig cells apoptosis through both genomic and nongenomic actions [58]. Recently, Annie and colleagues showed that dexamethasone affects testicular testosterone production by acting on Leydig cells via the modulation of PGC-1α and visfatin expression, both involved in gonadal steroidogenesis, while concomitantly impairing the differentiation of germ cells [59].

Together with tacrolimus, antimetabolites (in particular, mycophenolic acid) prevent the proliferation of T- and B-lymphocytes and represent the cornerstone of immunosuppressive therapy in KTRs. Although no data have demonstrated that antimetabolites may affect erectile function, some studies have reported that mycophenolic acid may negatively impact reproductive health. Indeed, it alters DNA synthesis through indirect mechanisms [60], leading to mutagenic and teratogenic effects [61]. However, several recent studies have suggested there was no significant increase in the number of congenital anomalies in children born from fathers who were treated with azathioprine or mycophenolate-mofetil [61,62]. There is very limited data on the impact of the more recent belatacept, rituximab, basiliximab, and anti-thymocyte globulin administration on sexual function and male fertility [61].

## 4. Treatment of ED in Kidney Transplant Recipients

A wide variety of treatments to restore erectile function are safe and effective in KTRs. PDE5 inhibitors (PDE5is) have been studied in these patients, and, among them, sildenafil [63,64,65,66] and vardenafil [67] have been used with success, showing no significant interactions with immunosuppressive drugs [68,69]. Additionally, psychosexual therapy, drug therapy, surgical treatment, transurethral or intracavernosal therapy, vacuum constriction devices, and low-intensity shockwave therapy (LiSWT) may be pursued. The detection of PDE5 expression in the cortex and inner medulla of human kidneys prompted the hypothesis of a possible natriuretic effect other than those exerted on penile vasculature itself. However, sildenafil administration in decompensated cirrhotic patients caused rapid activation of the renin–angiotensin–aldosterone system and sodium retention, which are associated with a decrease in arterial blood pressure [70]. Increased cyclic GMP levels have also been implicated in the control of renin secretion, and the administration of sildenafil exerted a stimulatory effect on renin secretion. This effect may help to explain why sildenafil has a minor effect on blood pressure, despite the widespread distribution of PDE5 in vascular tissues, and it is very safe on KTRs [71]. Sildenafil, the first available oral drug that has proven to be effective and safe for ED treatment in KTRs, as well as in patients undergoing hemodialysis, improves the penetration ability, maintenance frequency, and sexual domain scores (erectile function, sexual desire, intercourse, and overall satisfaction) without influencing the serum concentrations of cyclosporin [72]. Vardenafil is effective and well-tolerated in KTRs and shows similar efficacy and a similar safety profile as sildenafil [73]. The recommended starting dose of sildenafil in patients with severe kidney dysfunction (i.e., CLcr < 30 mL/min) should be 25 mg. Vardenafil 5 mg may be administered in patients with mild, moderate, or severe renal insufficiency [74]. The recommended starting dose of tadalafil, a PDE5i with a long half-life, is 5 mg in patients with moderate kidney insufficiency (CLcr 31–50 mL/minute), and the maximum total dose of 10 mg should not be exceeded in 48 h. For severely impaired patients (CLcr < 30 mL/min) and patients with ESRD undergoing dialysis, a 5-mg total dose in 72 h is recommended. Once-daily treatment is not recommended in patients with CLcr < 30 mL/minute [75] because of poor safety evidence related to KTRs and in patients undergoing dialysis.

As far as hormonal treatment goes, previous studies have found a positive correlation between the hormonal profile of KTRs and sexual function, confirming that the normalization of post-transplantation prolactin and testosterone levels yields several benefits, as well as shifting drug administration, as shown in Table 1 [76]. Interestingly, Chatterjee and colleagues reported that the administration of testosterone replacement therapy and sildenafil in KTRs with hypogonadism promotes an effective response, thereby underlining the importance of evaluating the gonadal function in KTRs with ED [77]. Testosterone exerts a crucial double role in male sexual function; it mediates the sexual desire by acting at the central nervous system level [78] and maintains penile PDE5 expression that triggers the NO cascade, leading to erection [79]. A growing body of evidence has reported that kidney transplantation promotes a significant rescuing of testosterone levels in a subset of KTRs, leading to improvement of ED [80,81]. Conversely, some authors have reported a high prevalence of the HPG axis abnormalities in male KTRs with well-functioning allografts [82]. Indeed, Lofaro and colleagues reported a high prevalence of hypogonadism in male KTRs [13].

Second-line treatments are penile injections and urethral suppositories. Intracavernous injections of alprostadil have been successfully used in immunosuppressed KTRs without any reports of kidney impairment or changes in CNI levels attributable to this treatment for ED [83]. Alprostadil can be usefully delivered by a urethral suppository, and the dosage required is about 25 times higher than that used for intracavernous injection.

Third-line therapy includes penile implants, which should be carefully considered in these patients, taking into account the following aspects: (i) stable graft function for at least 6 months; (ii) low doses of immunosuppressants; (iii) low probability of device malfunction; (iv) no intra-abdominal components to avoid confusion of the reservoir with the bladder in the event of subsequent kidney transplantation; (v) minimum tissue dissection; (vi) no skin or urinary tract infections; (vii) use of prophylactic antibacterials (parenteral, intraurethral, and topical); (viii) treatment with post-operative broad-spectrum oral antibacterials for 1–2 weeks [84].

Low-intensity shockwave therapy (Li-SWT) has recently emerged as a potential treatment for ED, and this has created considerable excitement due to its potential reparative mechanisms promoting the formation of new blood vessels and improving endothelial function in the corpora cavernosa. Yamaçake and colleagues have published promising results of the effects of six treatment sessions, with 7 out of 10 patients in the treatment group experiencing an improvement of at least 5 points in the IIEF-5 score vs. only 1 out of 10 in the sham group [85]. At first glance, this seems to confirm a possible effect of Li-SWT and suggests that the treatment could be offered specifically to KTRs. However, these results must be interpreted cautiously because of the very small number of patients treated.

A pioneering clinical experience evaluated the effectiveness of vacuum therapy in dialysis patients, with 73% achieving erection with the vacuum device. In this study, all hypogonadal men received testosterone therapy via implantation of depo-testosterone [86]. In the future, it will be appropriate to better understand the therapeutic potential of endothelial progenitor cells in these patients [87]. Their significance is certainly more solid from a diagnostic point of view as an early marker of endothelial damage. However, the intracavernous incorporation of these cells, in particular, the immunophenotypic variants with a higher degree of maturation, could represent an interesting challenge.

Although 38% of patients with testosterone deficiency display sexual symptoms, including ED, the latter does not usually correlate with the presence of hypogonadism in male KTRs [17]. Remarkably, it has been reported that in CKD patients, a 22% decrease in the risk of developing cardiovascular events for each nmol-per-liter increment in total testosterone concentration occurs [88]. Karakitsos and colleagues have linked CKD-related hypogonadism to precise clinical measures of endothelial dysfunction, reporting an inverse correlation between serum testosterone levels and intima/media thickness of the carotid artery and left ventricular mass index [89]. Furthermore, testosterone deficiency has been linked to a higher risk of all-cause mortality in male patients with CKD [90], with a more favorable overall prognosis seen in patients with higher testosterone levels undergoing hemodialysis [91]. Although transplantation may revert many of the consequences of renal insufficiency, this is not always the case for hypogonadism, probably because of the concomitant glucocorticoid and immunosuppressant use. Indeed, cyclosporin-A and tacrolimus exert a direct toxic effect on Leydig cells and the hypothalamic–pituitary–gonadal axis. Symptomatic hypogonadism is common in KTRs, as well as during dialysis, and testosterone replacement therapy can be safely prescribed even in the long-term, with significant improvements in anemia [91,92]. Testosterone pellets can be used in immunosuppressed KTRs without infectious complications [93]. Alternatively, the use of human chorionic gonadotropin may help to augment and increase endogenous testosterone levels, as well as the Leydig cell expression of CYP2R1, a key enzyme involved in vitamin D 25-hydroxylation, thus exerting dual protective effects [94].

## 5. Conclusions

The incidence of ED in patients with ERSD and KTRs is quite high, and its management is particularly difficult due to many interfering factors. The first approach should refer to changing the pharmacological interference scenario, especially for those drugs upsetting sexuality at the central (i.e., gonadotrophins, neurosteroids) and peripheral levels by shifting some adverse treatments to favorable ones whenever possible. The second approach may be to prescribe a PDE5i at a low dosage, considering that if testosterone-circulating levels are compatible with the diagnosis of hypogonadism, its efficacy may only be partial [80]. The possibility of using a combination of testosterone plus PDE5i should be considered, due to the beneficial effects on both systemic and local (penis, kidney) targets. Careful monitoring of KTRs is required both in terms of efficacy and safety because of the possible occurrence of treatment-emergent adverse events requiring drug withdrawal. Finally, topical or intracavernous injections of alprostadil in patients failing first- and second-line treatments are advised in the absence of contraindications. Penile implants should be regarded as third-line options arising from specific patient needs and compliance with the clinical conditions.

## Figures and Tables

**Table 1:** Factors and conditions influencing erectile dysfunction (ED) in chronic kidney disease (CKD)/kidney transplantation recipients (KTRs).

Factor/Condition	Evidence
Hormonal abnormalities	Hypergonadotropic hypogonadism: ↓ Total and free T = SHBG activity/levels ↑LH, ↑FSH Leydig/Sertoli cells function impairment Hypogonadotropic hypogonadism: ↑PRL→ ↓GnRH→ ↓LH → ↓T T deficit: ↓ sexual desire/erectile function corporal veno-occlusive dysfunction and fibrosis altered nitrergic activity and PDE5 expression ↑17ß-estradiol
Cardiovascular and endothelial dysfunction	Direct impact: ↓Endothelial function→↓NO→ED Indirect impact: MS→ CVD→ ED
Hypercholesterolemia	Endothelial dysfunction→ ↓NO→ ↓ L-arginine synthesis/availability →vasculogenic ED Negative impact on penile hemodynamics
Nervous system abnormalities	Autonomic neuropathy secondary to DM or CKD Peripheral neuropathy secondary to DM or CKD Polyneuropathy
Vitamin D deficiency/hyperparathyroidism	↓1,25 (OH) Vitamin D→↑ PTH ↑ PTH→↑ PRL→ ↑ED
Zinc depletion	Direct zinc removal during hemodialysis/↓GI zinc absorption→↓T→ED EPO resistant anemia Altered hematopoietic progenitor cell development Sometimes no reversal ED after zinc supplementation
Anemia	↓ EPO→ normocytic normochromic anemia ↓EPO→ ↓T, ↑PRL ↓EPO→ ↓ neuroprotective, antiapoptotic and angiogenic effects of EPO Anemia→ hypoxia→ ↓NO synthase activity, ↑ increased collagen synthesis→ ↓ relaxation of corpus cavernosum smooth muscle cells→ ED
Age	Direct correlation with prevalence and gravity of ED
Education	Inverse correlation with ED
Voluptuous habits	Role still unclear Detrimental effect of smoking on erectile function No correlation between smoking and ED in KTR in some studies
Duration time of dialytic treatment	Direct correlation with penile morphological and hemodynamic abnormalities Greater improvement of ED in KTR underwent to dialysis for 6 months No improvement of ED in KTR underwent to dialysis for 6–24 months Effect of early transplantation in the prevention/reverse of penile vasculopathy or sex hormones abnormalities
Drugs	Antihypertensive, thiazides, aldosterone receptor blockers, and beta-adrenergic receptor blockers, cimetidine, tricyclic antidepressants, metoclopramide→ onset/exacerbation of ED Calcineurin inhibitors (cyclosporin A, tacrolimus) → maintenance of the endothelial and gonadic dysfunction previously determined by uremiam TOR inhibitors/corticosteroids→ impact on testicular function and/or structure >1 antihypertensive drug administration→ ↑ prevalence of ED Association between ACEI/ARBs and DE
Surgical procedures	Interruption of arteria iliac distal end→ blood flow→ ED ED in end-to-end anastomosis to the internal iliac artery > ED end-to-side anastomosis to the external iliac artery Slight ↓in penile arterial inflow in KTR onto external iliac artery→ ED Iterative transplantation→ end-to-side anastomosis on internal iliac artery→ penile hypoafflux→ ED
Psychosocial factors	Depression, anxiety, worrying, ↓health-related quality of life→ ED Altered body’s image perception→ ED and sexual function Somatic, relational and psychosocial comorbidities

ED: erectile dysfunction; KTR: kidney transplantation recipient; ↑: increased; ↓: decreased; =: unchanged; T: testosterone; SHBG: Sex Hormone Binding Globulin; LH: luteinizing hormone; FSH: follicle-stimulant hormone; GnRH: gonadotropin-releasing hormone; PRL: prolactin; PDE5: phosphodiesterase-5; NO: nitric oxide; MS: metabolic syndrome; CVD: cardiovascular disease; DM: diabetes mellitus; CKD: chronic kidney disease; PTH: parathyroid hormone; EPO: erythropoietin; ACEI: angiotensin-converting-enzyme (ACE) inhibitors; ARBs: angiotensin II receptor blockers.
